# *Notes from the Field:* Human *Brucella abortus* RB51 Infections Caused by Consumption of Unpasteurized Domestic Dairy Products — United States, 2017–2019

**DOI:** 10.15585/mmwr.mm6807a6

**Published:** 2019-02-22

**Authors:** María E. Negrón, Grishma A. Kharod, William A. Bower, Henry Walke

**Affiliations:** 1 Division of High-Consequence Pathogens and Pathology, National Center for Emerging and Zoonotic Infectious Diseases, CDC.

Since August 2017, CDC has confirmed three cases of brucellosis attributed to *Brucella abortus* cattle vaccine strain RB51 (RB51). Each case was associated with consumption of domestically acquired unpasteurized (raw) milk products (*1*). Patient symptoms varied and included fever, headache, overall malaise, and respiratory symptoms. In total, at least eight persons met the probable case definition of a clinically compatible illness epidemiologically linked to a shared contaminated source (*2*). In addition, hundreds of persons, from dozens of states, were potentially exposed to the contaminated raw milk products (*3*).

Consumption of raw milk products increases the risk for infection with pathogens such as *Escherichia coli*, *Campylobacter*, *Listeria*, and *Brucella* spp. Raw milk–related disease outbreaks occur more often in states with legalized raw milk sales (*4*). Approximately 75% of U.S. states have laws allowing various types of raw milk sales (*5*).

Brucellosis, caused by *Brucella* spp., is primarily an animal disease; however, exposure to infected animals or raw milk products can cause human disease. In humans, brucellosis is characterized by nonspecific symptoms, including fever, arthralgia, myalgia, and sweats; miscarriage and other sequelae can occur. Human brucellosis is rare in the United States, with 80–120 cases reported annually; most of these are associated with *Brucella* exposures abroad (CDC, unpublished data, 2019). The rarity of human brucellosis in the United States is mainly attributable to pasteurization and the successful U.S. State-Federal Cattle Brucellosis Eradication Program. As a result of the program’s focus on disease surveillance and cattle vaccination, *B. abortus* in livestock has been eliminated, except in limited areas where disease reintroduction from infected wildlife occurs.

RB51 is a live, attenuated vaccine that has been used to vaccinate cattle against *B. abortus* in the United States since 1996. Although rare, it is possible for cattle to shed RB51 in their milk, even when vaccine label recommendations are followed (*6*). Consuming this raw milk can cause human infections, which, unlike infections caused by field *Brucella* strains, do not stimulate an antibody response detectable by commercially available serological assays and can be missed by tests normally used for diagnosis. In addition, RB51 is resistant to rifampin, a first-line antibiotic used to treat human brucellosis (*3*). When evaluating patients whose symptoms are consistent with brucellosis, clinicians should consider RB51 infection and inquire about raw milk consumption as part of the patient’s exposure history (*3*).

Several actions could be considered to reduce the risk for raw milk–related RB51 human infections. CDC recommends that public health and regulatory authorities continue supporting pasteurization and consider further restricting the sale and distribution of raw milk and raw milk products in their jurisdictions.* States might explore options such as the United States Animal Health Association’s recommendations that state animal health officials and cattle industry representatives evaluate the need for the RB51 vaccine in areas where *B. abortus* is not endemic in wildlife (*7*). Modifying current RB51 vaccine labels to include information about possible shedding in milk could also improve awareness. Finally, veterinarians and dairy farm owners need to be aware that RB51 vaccination might pose a risk when given to cows whose milk is intended to be consumed unpasteurized.
